# Three-Dimensional Aspects of the Lingual Papillae and Their Connective Tissue Cores in the Tongue of Rats: A Scanning Electron Microscope Study

**DOI:** 10.1155/2014/841879

**Published:** 2014-11-10

**Authors:** Gabriela de Souza Reginato, Cristina de Sousa Bolina, Ii-sei Watanabe, Adriano Polican Ciena

**Affiliations:** ^1^Departamento de Cirurgia, Faculdade de Medicina Veterinária e Zootecnia da Universidade de São Paulo, Avenida Prof. Dr. Orlando Marques de Paiva n. 87, Cidade Universitária, 05508-270 São Paulo, SP, Brazil; ^2^Departamento de Anatomia, Instituto de Ciências Biomédicas (ICB-III), Avenida Prof. Lineu Prestes n. 2415, Cidade Universitária, Butantã, 05508-900 São Paulo, SP, Brazil; ^3^Laboratório de Morfologia e Atividade Física, Instituto de Biociências, Universidade Estadual Paulista (UNESP), Avenida 24 A n. 1515, Bela Vista, 13506-900 Rio Claro, SP, Brazil

## Abstract

The aim of the present study was to describe the tridimensional morphological characteristics of the lingual papillae and their connective tissue cores (CTCs) in Sprague Dawley rats. Four types of papillae were reported on the dorsal surface. Filiform papillae were distributed on the tongue surface and after epithelial maceration a conic and multifilamentary shape of the CTCs was revealed. Fungiform papillae were reported on the rostral and middle regions covered by a squamous epithelium. After the removal of the epithelium, the shape of a volcano with the taste orifice at its top was noted. Foliate papillae were composed of five pairs of epithelial folds situated on the lateral-caudal margin of the tongue. After the removal of the epithelium, they were shown to be limited by thin laminar projections. The vallate papilla with an oval shape was present in the caudal region and delimited by an incomplete groove. The morphological characteristics of the lingual papillae of Sprague Dowley rats, three-dimensional SEM images, and the types of papillae on the dorsal surface were similar to those reported previously in other rodent mammals. The maceration technique revealed the details of extracellular matrix with varied shapes form of connective tissue cores.

## 1. Introduction

The tongue fills most of the oral cavity and extends itself to the mouth pharynx. Its root is linked to a body and a free apex. It is in fact a muscular organ capable of vigorous and precise movements, such as holding, lapping, grooming and manipulation of food within the oral cavity, and vocalization [1].

The body of the tongue consists of a mass of bundles interwoven with varied disposition of skeletal striated muscular fibers which permit a great variation of tongue movements. The fibers may be classified into two groups: fibers that originate outside of the tongue (extrinsic muscles) and those that originate within the tongue and which are inserted into it. The latter form the intrinsic muscles that change the tongue's shape [2].

The tongue exhibits a lining or rather a continuous mucous of different thicknesses, throughout its surface. The epithelium is thick and rigid on the dorsal surface where tongue wear is greater due to friction with food [3]. The ultrastructural elements of the epithelium vary in the morphology of different mammals and show several types of papillae. Besides being associated with animal species, variations may be also related to type of food and to the animal's adaptation to environmental conditions [4].

The lingual epithelium is made up of four types of tongue papillae, namely, filiform, fungiform, foliate, and vallate papillae, featuring mechanical and taste functions [5]. However, shape, size, and organization of the tongue papillae vary according to each mammal species [6].

The filiform papillae are distributed thickly on all the dorsal and lateral surfaces of the tongue. They assist in the manipulation of food and increase friction during chewing. They are predominant on the apex and generally are inclined at the caudal region, whereas they are modified and presented various shapes at the root [7]. Differences in the morphology of filiform papillae may be observed on the apex and in the middle region of tongues of several species. The conical papillae are elongated shaped with a wide base and flat apex. Their lining consists of well-developed keratinized stratified epithelial tissues with abrasion and protection as their main role [8]. The fungiform papillae may be identified on the apex, featuring a great quantity of connective tissues and adjacent epithelial layer. They are well vascularized but in a lesser number than the filiform papillae, taking the shape of a “mushroom.” They may present taste buds generally situated on the top of the papillae and are involved in the sensorial system related to taste [9].

The foliate papillae when present are situated at the bilateral margins of the caudal region, are leaf-shaped structures, separated from one another by an invagination of the mucous membrane, and have taste buds. The amount of foliate papillae may vary according to the evolution of each species [10].

The vallate papilla is the biggest of the papillae and is situated on the caudal region of the dorsal surface, involved in a deep continuous groove. The quantity and shape of the vallate papillae vary widely and depend on the species analyzed [11]. They vary in size and shape, from round shaped to flat shaped, either lying parallel or in rows at each side of the caudal region. Groups of glands, also known as von Ebner glands, are detected in the lower part of the vallate papillae. They are salivary serous glands where ducts open at the base of the papillae grooves and secrete a watery liquid that dissolves food contents and makes easy taste perception [12]. In the order Rodentia, several studies have been undertaken on mice [13, 14], cavy [15], agouti [12], flying squirrel [16], American beaver [17], and lowland paca [7]. However, studying the lingual papillae of rats may note different shapes on the surface and their connective tissue cores (CTCs) in the treated samples with NaOH solution. The types and subtypes of tongue's papillae of rodent mammals may exhibit several differences in the morphological characteristics to classification, shape, and taste buds. The aim of the present study was to describe the three-dimensional morphological characteristics of the lingual papillae and their connective tissue cores (CTCs) of Sprague Dawley rats employing scanning electron microscope methods.

## 2. Material and Methods

Four tongues of four-month-old male rats, species, order: Rodentia, were investigated. The animals were kept in polypropylene cages with water and ration “ad libitum,” maintained in 12-hour-light/dark periods, at a mean temperature of 25 ± 2°C. This study was approved by the Committee for Ethics in the Use of Animals (CEUA) of the Institute of Biomedical Studies of the University of São Paulo, Brazil (159/2010).

### 2.1. Scanning Electron Microscopy

The tongues (*n* = 4) were immersed in modified Karnovsky fixative solution according to Watanabe and Yamada method [18]. The samples were then washed in a buffer solution and divided for the different techniques. Conventional technique comprised the washing of samples in distilled water for 2 hours at room temperature to analyze the epithelium surface. For maceration, the other samples were washed in distilled water and immersed in a 10% sodium hydroxide (NaOH) aqueous solution for 4 days at room temperature [19–21] for the removal of the epithelial surface and the analysis of the connective tissue core (CTC). They were then washed in distilled water, with frequent changes, for two days, at 4°C. After this stage, all samples were dehydrated in an increasing series of alcohols and dried in a critical point dryer (Balzers CPD-030) with liquid CO2. Samples were mounted on a metallic base, coated with gold ions (Balzers-040 SDC) [22], and examined using a scanning electron microscope LEO 435 VP.

## 3. Results

**Figure 1:**
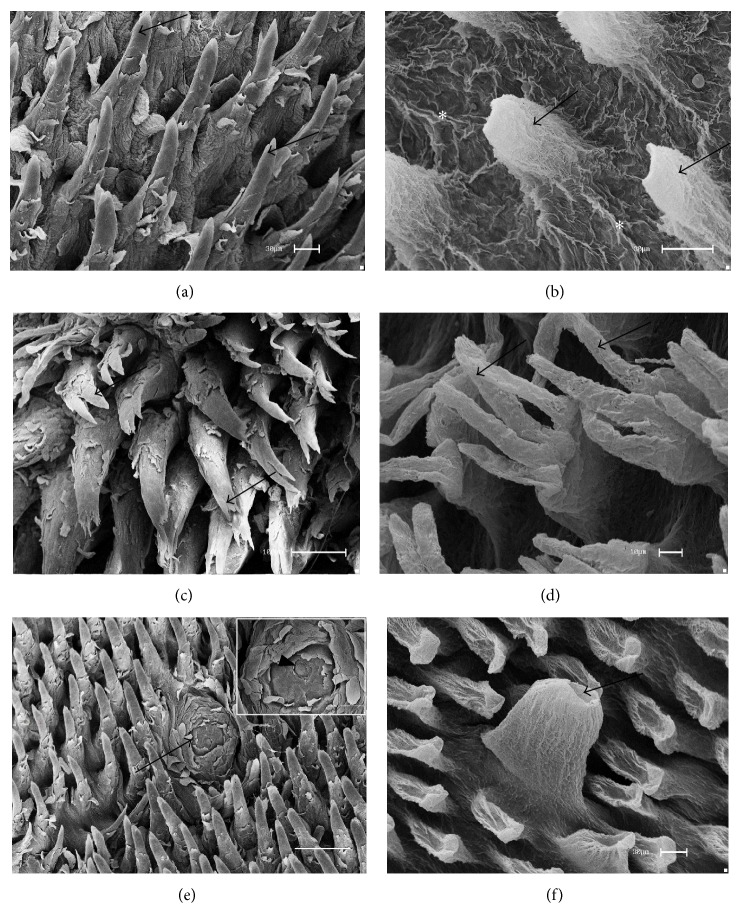
Scanning electron micrographs—filiform and fungiform papillae of rats. (a) Conical filiform (arrows) and (b) after maceration with NaOH the CTCs of the filiform papillae presented conical (arrows) and bundles (∗) in the interpapillary zone. (c) Multifilamentary papillae (arrows) and (d) their CTCs revealed three to four filaments (arrows) in the third upper part. (e) The fungiform papillae (arrow) exhibited dome-like shape between conical filiform papillae and the highlighted taste orifice (arrowhead) may be seen in the surface. (f) After the removal of the epithelium, the CTCs of the fungiform papillae revealed volcano-like shape, with a cavity on the top for the taste buds (arrow). Bars: 30 *μ*m (a, b, f), 100 *μ*m (c, e), and 10 *μ*m (d).

The analyses by scanning electron microscope showed that filiform papillae were the most numerous on the dorsal surface of the tongue with a decreased amount on the caudal region. They are shaped and have different diameters according to the region. In the rostral region the papillae are low cone shaped, with a rounded apex (Figure 1(a)). Elongated and multifilamentary papillae may be found in the middle and caudal regions, with their respective long and thin filaments (Figure 1(c)). After the removal of the epithelium, the CTCs of the filiform papillae (rostral region) revealed low, with a cone-shaped apex, a base wider than the apex, and thin bundles in the interpapillae zone (Figure 1(b)), different from other filiform papillae. A greater magnification revealed three to four filaments (multifilamentary) starting from the third mid-upper part of the CTCs (Figure 1(d)). Fungiform papillae were distributed in the rostral and middle regions of the dorsal surface. They are very numerous in the rostral region, with a rounded shape and dome-like top. In the details one may note that the papillae surface comprises a squamous epithelium and a taste orifice on the top of the surface (Figure 1(e)). After the epithelium removal, the volcano shape was revealed and a cavity for the taste bud on the papillae top was observed (Figure 1(f)).

**Figure 2:**
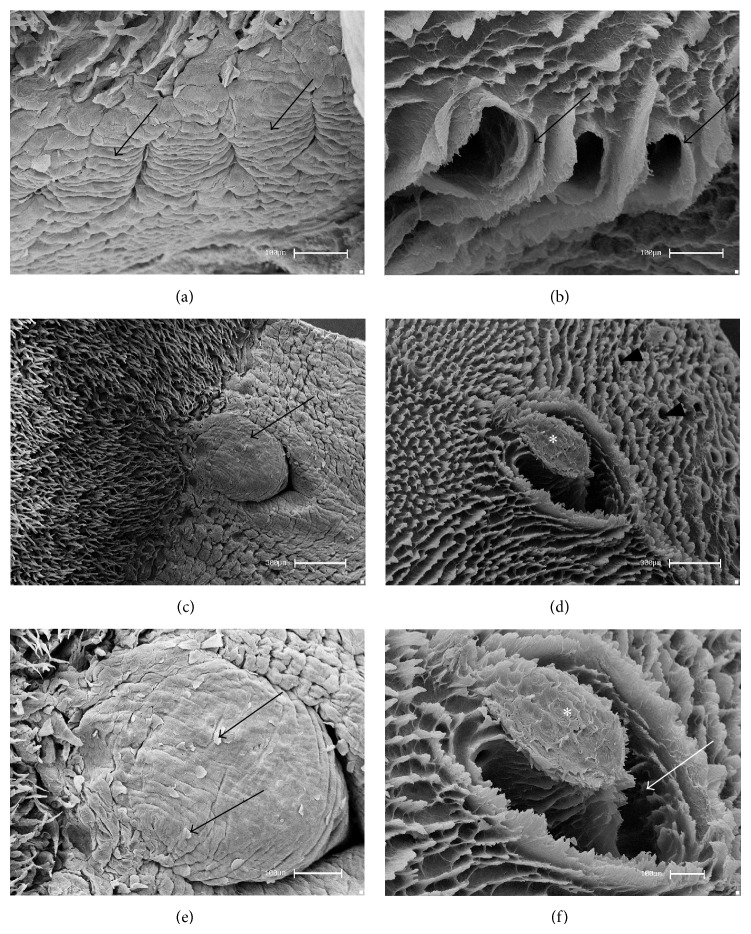
Scanning electron micrographs—foliate and vallate papillae of rats. (a) The foliate papillae are constituted by epithelial folds (arrows) and separated by parallel grooves. (b) After the removal of the epithelial tissue, the CTCs of the foliate papillae showed wide grooves limited by laminar projections (arrows). (c) The vallate papilla (arrow) is situated in the caudal region and (d) after the removal of the epithelium the general aspects of the CTCs (∗), delimited by CTCs of the filiform papillae and salivary glands ducts (arrowheads), were observed. (e) At higher magnification, squamous epithelium (arrows) in the surface was noted. (f) At higher magnification the constitution of the CTC by a thick bundle (∗) margined by a wide groove (arrow) was revealed. Bars: 100 *μ*m (a, b, e, f) and 300 *μ*m (c, d).

The foliate papillae presented five pairs of epithelial folds, separated by parallel grooves on the lateral-caudal margin of the tongue (Figure 2(a)). After the removal of the epithelium, CTCs with wide oval grooves, limited by laminar projections, were observed (Figure 2(b)). The vallate papilla is situated in the middle of the caudal region, with an oval shape, wrapped by an incomplete groove on the hind part (Figure 2(c)). A greater magnification showed that the papillae are lined by a squamous epithelium (Figure 2(e)). After the removal of the epithelium the CTC constitution by a thick bundle margined by a wide groove was observed. The aperture of salivary ducts was seen on the side walls (Figures 2(d) and 2(f)).

## 4. Discussion

The results obtained in this study revealed mainly the three-dimensional characteristics of the tongue's papillae and the original disposition of connective tissue cores (CTCs) in the Sprague Dawley rats. In these data four types of papillae, filiform, fungiform, foliate, and vallate, were observed on the dorsal surface of the lingual epithelial layer similar to those reported by Ciena et al. [12].

The filiform papillae presented low cone-shaped in the rostral region although the papillae were elongated and were multifilamentary in the middle and caudal regions. After the removal of the epithelium, the CTCs of the filiform papillae were low, with a cone-shaped apex in the rostral region. However, multifilamentary papillae revealed three to four filaments in the middle and caudal regions. Each cylinder-shaped filament lay on the upper section, starting from the mid-third part of the connective papillae. According to Kilinc et al. [9], the filiform papillae are the most abundant on the tongue of rodent mammals, distributed throughout the dorsal epithelium surface and decreased in the caudal region [12]. The morphological characteristics of the filiform papillae of the rats differed from those of other rodents as has been reported in the flying squirrel. Emura et al. [16] reported that the latter species presented conical papillae in the caudal region and elongated papillae in the rostral region. These morphological characteristics were similar to those of the filiform papillae reported in the cavy's tongue, as described by Watanabe et al. [15], and in the Patagonian cavy's tongue, as described by Emura et al. [23].

The fungiform papillae were distributed in the rostral and middle regions of the dorsal surface. Reports were similar to descriptions in the tongue of other rodents such as the agouti [12], blind mole rat [9], and paca [7]. After the epithelial removal, the shape of a volcano was revealed and a cavity was seen on the papillae top, the probable site of the taste buds, similar to those reported on the agouti's tongue [12]. Current literature shows that other terms are used to describe the different shapes of the fungiform papillae, such as “flower bud” in the Patagonian cavy [23] and “fist” in the American castor [17] and the guinea pig [24].

The foliate papillae revealed five epithelium folds separated by parallel grooves on the lateral-caudal margin of the tongue. After the removal of the epithelium, wide oval grooves limited by laminar projections were observed. The localization of such papillae was similar to that in the paca [7], flying squirrel [16], and cavy [15]. According to Emura et al. [23], foliate papillae in Rodentia are well developed. Further, the number of epithelial folds may vary according to each species. There were 12 pairs of well-designed foliate papillae in the tongue of the agouti [12] and between 22 and 25 pairs in the tongue of the American castor [17].

A vallate papilla was observed in the central caudal region, limited by an incomplete groove at the upper part. After the removal of the epithelium, the constitution of a thick bundle margined by a wide groove of the CTC was noted. The quantity and the shape of these papillae differed according to the species. The number of papillae in the bank vole [25] is similar to that in the rat, with only one papilla. Further, the paca [7], Patagonian cavy [23], and blind mole rat [9] present a pair of parallel papillae, whereas the flying squirrel has three papillae [16]. However, four papillae were reported in the agouti, the largest amount in rodents as described by Ciena et al. [12].

## 5. Conclusion

The morphological characteristics of the lingual papillae of Sprague Dawley rats, three-dimensional SEM images, and the types of papillae on the dorsal surface were similar to those reported previously in other rodent mammals. The maceration technique revealed the details of extracellular matrix with varied shapes form of connective tissue cores.
